# Supplementary material for: Molecular Cloning and Exploration of the Biochemical and Functional Analysis of Recombinant Glucose-6-Phosphate Dehydrogenase from Gluconoacetobacter diazotrophicus PAL5
Source: Int J Mol Sci. 2019 Oct 24;20(21):5279. doi: 10.3390/ijms20215279 (PMC6862599; doi:10.3390/ijms20215279)
Supplement: Supplementary file 1 [file ijms-20-05279-s001.zip › Supplementary files/Supplementary File.docx]

**Supplementary file**

**Figure S1**. Multiple alignments of the amino acid sequences. Multiple sequence alignment was performed with MAFFT V7.427 [15] (file: zwf_mafft.aln.fasta) and visualized with the online program Jalview [16]. Three fully conserved fragments are shown as colored boxes. A total of 60 amino acid sequences for G6PD protein were obtained from Swiss-Prot and the National Center for Biotechnology Information (NCBI) (Table S1).


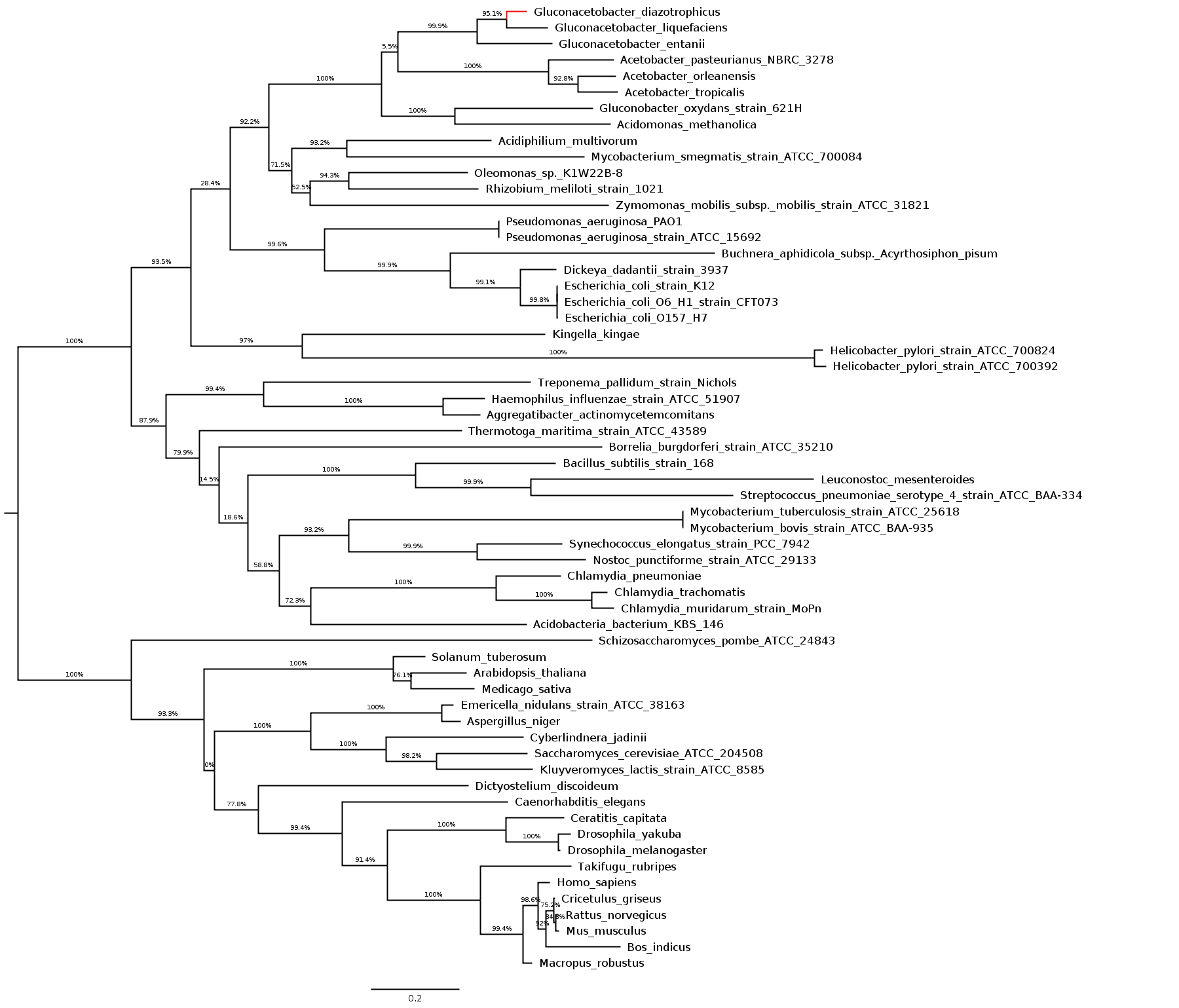


**Figure S2.** Molecular phylogenetic analysis based on the different G6PD protein sequences showed a phylogenetic relationship between *Gluconacetobacter diazotrophicus* PAL5 (GDI) and related members of the genus *Gluconacetobacter*. The bar (0.2) indicates the genetic distance. A total of 60 amino acid sequences for the G6PD protein were obtained from Swiss-Prot and NCBI (Table S1). The sequences were aligned with the program with MAFFT v7.427 [15] to generate a phylogenetic tree and was constructed with FastTree 2.1.10 [52] using the Whelan and Goldman model and 1,000 bootstraps (file: zwf_mafft.tree).

**Figure S3.** Homology model of the G6PD protein of GDI. (A) Dimeric structure of the G6PD protein of GDI. In one monomer, the enzyme is colored in medium see green; the other monomer is steel blue (file: ZWF model). Note that the G6PD active site of one monomer (medium see green) showed the conserved sequences GxGGDLT (residues 31-37, dark orange), EKPxG (residues 169-173, navy blue) and RIDHYLGKE (residues 197-205, green yellow). (B) Structural alignment of the human G6PD enzyme (PDB entry 2BH9; gold), G6PD from *L. mesenteroides* **(**PDB entry 1H9A; indigo) and the minimized model of the G6PD from GDI (medium see green). The catalytic NADP^+^ is shown in red as a molecular surface representation, and structural NADP^+^ from human G6PD is shown in dark green, also with molecular surface representation of the same color. (C) The G6PD active site shows the representative residues (T37, P171, and K204) of these conserved sequences as black cylinders. The catalytic NADP^+^ and G6P substrates are shown as red and royal blue molecular surface representations, respectively. Structural alignment in Figure B was performed using Coot [56].

**Table S1.** Comparative analysis of G6PDs region protein sequence of *G. diazotrophicus* PAL5 and the G6PDs proteins from other species with HHblits.

| **Hit** | **Species** | **Probability** | **E-value** | **Aligned Cols** | **Target Lenght** | **Score** | **Identities** | **Similarity** |
| --- | --- | --- | --- | --- | --- | --- | --- | --- |
| [A0A0D6ML00](http://www.uniprot.org/uniprot/A0A0D6ML00) | *Tanticharoenia sakaeratensis* | 100 | 2.70E-177 | 493 | 511 | 1310.35 | 65 % | 1.01 |
| [A0A2A2YE23](http://www.uniprot.org/uniprot/A0A2A2YE23) | *Verrucomicrobiae bacterium* | 100 | 3.70E-142 | 484 | 510 | 1059.61 | 53 | 0.939 |
| [A0A2S7DNS6](http://www.uniprot.org/uniprot/A0A2S7DNS6) | *Xanthomonas cucurbitae* | 100 | 2.80E-141 | 488 | 652 | 1056.34 | 54 | 0.967 |
| [A0A2V6R8R5](http://www.uniprot.org/uniprot/A0A2V6R8R5) | *Rokubacteria bacterium* | 100 | 1.60E-138 | 485 | 503 | 1030.88 | 55 | 0.96 |
| [A0A2W5UV98](http://www.uniprot.org/uniprot/A0A2W5UV98) | *Citrobacter freundii* | 100 | 6.10E-134 | 481 | 509 | 997.89 | 54 | 0.941 |
| [A0A2M8PAT3](http://www.uniprot.org/uniprot/A0A2M8PAT3) | *Chloroflexi bacterium* | 100 | 3.30E-133 | 471 | 483 | 990.33 | 55 | 0.91 |
| [A0A2D7G1V4](http://www.uniprot.org/uniprot/A0A2D7G1V4) | *Gammaproteobacteria bacterium* | 100 | 2.20E-127 | 486 | 656 | 967.21 | 49 | 0.905 |
| [A0A2V6XG61](http://www.uniprot.org/uniprot/A0A2V6XG61) | *Candidatus* Rokubacteria | 100 | 5.60E-127 | 480 | 617 | 962.05 | 52 | 0.922 |
| [A0A0F3IRN7](http://www.uniprot.org/uniprot/A0A0F3IRN7) | *Elstera litoralis* | 100 | 2.40E-124 | 474 | 489 | 928.99 | 49 | 0.83 |
| [A0A271J0P7](http://www.uniprot.org/uniprot/A0A271J0P7) | *Rubrivirga marina* | 100 | 3.70E-123 | 466 | 488 | 918.59 | 50 | 0.853 |
| [A4ENV5](http://www.uniprot.org/uniprot/A4ENV5) | *Roseobacter sp* | 100 | 4.50E-123 | 474 | 496 | 921.37 | 48 | 0.854 |
| [A0A260EE74](http://www.uniprot.org/uniprot/A0A260EE74) | *Rhodococcus sp.* | 100 | 1.10E-121 | 480 | 516 | 911.53 | 49 | 0.864 |
| [A0A2G2QFM6](http://www.uniprot.org/uniprot/A0A2G2QFM6) | *Methylophaga sp.* | 100 | 3.00E-121 | 475 | 489 | 906.38 | 47% | 0.836 |
| [A0A1M5RSH1](http://www.uniprot.org/uniprot/A0A1M5RSH1) | *Hydrocarboniphaga daqingensis* | 100 | 3.30E-120 | 476 | 497 | 901.15 | 48% | 0.844 |
| [D2U859](http://www.uniprot.org/uniprot/D2U859) | *Xanthomonas albilineans* | 100 | 4.10E-118 | 487 | 663 | 887.93 | 54% | 0.951 |
| [A0A1W4U5C2](http://www.uniprot.org/uniprot/A0A1W4U5C2) | *Drosophila ficusphila* | 100 | 4.10E-114 | 370 | 394 | 836.73 | 61% | 1.066 |
| [A0A2E2UQM5](http://www.uniprot.org/uniprot/A0A2E2UQM5) | *Trueperaceae bacterium* | 100 | 1.90E-112 | 474 | 479 | 843.29 | 48% | 0.842 |
| [A0A2V9Q6G7](http://www.uniprot.org/uniprot/A0A2V9Q6G7) | *Acidobacteria bacterium* | 100 | 9.60E-110 | 472 | 839 | 835.07 | 56% | 0.992 |
| [A0A1H7Z6Q8](http://www.uniprot.org/uniprot/A0A1H7Z6Q8) | *Lactobacillus ruminis* | 100 | 2.70E-109 | 467 | 487 | 821.99 | 45% | 0.781 |
| [A0A2N6BTK0](http://www.uniprot.org/uniprot/A0A2N6BTK0) | *Deltaproteobacteria bacterium* | 100 | 5.70E-108 | 380 | 401 | 797.33 | 54% | 0.899 |
| [A0A136P0P3](http://www.uniprot.org/uniprot/A0A136P0P3) | *Chlorobi bacterium* | 100 | 4.50E-108 | 379 | 395 | 796.77 | 54% | 0.927 |
| [A0A0P9FAB3](http://www.uniprot.org/uniprot/A0A0P9FAB3) | *Kouleothrix aurantiaca* | 100 | 8.30E-106 | 313 | 318 |  | 62% | 1.024 |
| [A0A2N7VK94](http://www.uniprot.org/uniprot/A0A2N7VK94) | *Paraburkholderia soli* | 100 | 9.40E-105 | 450 | 461 | 786.26 | 46% | 0.823 |
| [A0A1G2MW33](http://www.uniprot.org/uniprot/A0A1G2MW33) | *Taylorbacteria bacterium* | 100 | 1.30E-10 | 457 | 478 | 787.47 | 47% | 0.817 |
| [V9GEQ3](http://www.uniprot.org/uniprot/V9GEQ3) | *Paenibacillus sp.* | 100 | 2.10E-104 | 480 | 512 | 787.25 | 42% | 0.758 |
| [E0E4Y8](http://www.uniprot.org/uniprot/E0E4Y8) | *Peptostreptococcus stomatis* | 100 | 1.90E-102 | 443 | 457 | 769.07 | 47% | 0.789 |
| [A0A1G2X8A6](http://www.uniprot.org/uniprot/A0A1G2X8A6) | *Planctomycetes bacterium* | 100 | 3.80E-98 | 453 | 579 | 744.24 | 49% | 0.877 |
| [A0A1F6IVI0](http://www.uniprot.org/uniprot/A0A1F6IVI0) | *Candidatus* Levybacteria | 100 | 7.70E-95 | 468 | 491 | 719.49 | 40% | 0.725 |
| [S4XJ39](http://www.uniprot.org/uniprot/S4XJ39) | *Sorangium cellulosum* | 100 | 3.70E-94 | 473 | 677 | 714.8 | 52% | 0.899 |
| [A0A2W4P3M9](http://www.uniprot.org/uniprot/A0A2W4P3M9) | *Chloroflexi bacterium* | 100 | 5.10E-93 | 313 | 335 | 683.42 | 54% | 0.929 |
| [A0A2A9E6X3](http://www.uniprot.org/uniprot/A0A2A9E6X3) | *Sanguibacter antarcticus* | 100 | 1.10E-90 | 458 | 506 | 691.48 | 43% | 0.726 |
| [A0A0J9XHW7](http://www.uniprot.org/uniprot/A0A0J9XHW7) | *Geotrichum candidum* | 100 | 2.60E-90 | 469 | 519 | 691.02 | 39% | 0.679 |
| [A0A1Q7XDF5](http://www.uniprot.org/uniprot/A0A1Q7XDF5) | *Cyanobacteria bacterium* | 100 | 2.00E-86 | 318 | 329 | 637.03 | 53% | 0.901 |
| [D2V362](http://www.uniprot.org/uniprot/D2V362) | *Naegleria gruberi* | 100 | 3.70E-85 | 472 | 550 | 657.33 | 42% | 0.757 |
| [A0A2V3DMZ0](http://www.uniprot.org/uniprot/A0A2V3DMZ0) | *Arthrobacter psychrochitiniphilus* | 100 | 9.20E-85 | 463 | 497 | 649.04 | 38% | 0.632 |
| [A0A097AR31](http://www.uniprot.org/uniprot/A0A097AR31) | *Thermoanaerobacter kivui* | 100 | 2.50E-84 | 433 | 492 | 640.62 | 45% | 0.824 |
| [A2G0T4](http://www.uniprot.org/uniprot/A2G0T4) | *Trichomonas vaginalis* | 100 | 9.70E-84 | 451 | 706 | 656.99 | 41% | 0.751 |
| [A0A1D6ACH9](http://www.uniprot.org/uniprot/A0A1D6ACH9) | *Triticum aestivum* | 100 | 2.40E-82 | 461 | 593 | 639.47 | 40% | 0.689 |
| [Q057M8](http://www.uniprot.org/uniprot/Q057M8) | *Buchnera aphidicola* | 100 | 4.20E-82 | 473 | 486 | 627.44 | 40% | 0.756 |
| [M7MW36](http://www.uniprot.org/uniprot/M7MW36) | *Cesiribacter andamanensis* | 100 | 3.40E-81 | 395 | 448 | 610.34 | 47% | 0.8 |
| [A0A2V3IP90](http://www.uniprot.org/uniprot/A0A2V3IP90) | *Gracilariopsis chorda* | 100 | 1.30E-79 | 476 | 601 | 618.84 | 39% | 0.652 |
| [Q867J3](http://www.uniprot.org/uniprot/Q867J3) | *Acraea encedon* | 100 | 7.00E-79 | 392 | 411 | 597.29 | 41% | 0.714 |
| [A0A258CA21](http://www.uniprot.org/uniprot/A0A258CA21) | *Pseudomonadales bacterium* | 100 | 1.60E-77 | 373 | 384 | 581 | 44% | 0.777 |
| [A0A2N2JFK6](http://www.uniprot.org/uniprot/A0A2N2JFK6) | *Deltaproteobacteria bacterium* | 100 | 3.20E-77 | 473 | 759 | 598.68 | 46% | 0.829 |
| [A0A0M3QZH8](http://www.uniprot.org/uniprot/A0A0M3QZH8) | *Drosophila busckii* | 100 | 2.80E-77 | 395 | 493 | 594.75 | 43% | 0.728 |
| [A0A2H0RH92](http://www.uniprot.org/uniprot/A0A2H0RH92) | *Candidatus* Vogelbacteria | 100 | 3.10E-76 | 343 | 365 | 572.72 | 41% | 0.763 |
| [A0A1G2KS17](http://www.uniprot.org/uniprot/A0A1G2KS17) | *Candidatus* Sungbacteria | 100 | 3.00E-76 | 453 | 790 | 610.32 | 39% | 0.714 |
| [A0A2K1JVX0](http://www.uniprot.org/uniprot/A0A2K1JVX0) | *Physcomitrella patens* | 100 | 7.30E-76 | 478 | 612 | 591.64 | 39% | 0.686 |
| [M8CKL4](http://www.uniprot.org/uniprot/M8CKL4) | *Aegilops tauschii* | 100 | 2.20E-75 | 456 | 597 | 587.19 | 39% | 0.697 |
| [A0A158NQV6](http://www.uniprot.org/uniprot/A0A158NQV6) | *Atta cephalotes* | 100 | 1.60E-74 | 473 | 630 | 583.79 | 38% | 0.662 |

**Table S2.** G6PD protein sequences used in the multiple alignment.

| **Species** | **Access Code** | **Database** |
| --- | --- | --- |
| *Gluconacetobacter diazotrophicus* | WP_012554804.1 | NCBI |
| *Pseudomonas aeruginosa* PAO1 | AAC38311.1 | NCBI |
| *Acidobacteria bacterium* KBS 146 | WP_026388357.1 | NCBI |
| *Kingella kingae* | SMC15651.1 | NCBI |
| *Acetobacter orleanensis* | GEB82938.1 | NCBI |
| *Acetobacter pasteurianus* NBRC 3278 | GCD62654.1 | NCBI |
| *Acetobacter tropicalis* | WP_086640208.1 | NCBI |
| *Gluconacetobacter entanii* | PYD62955.1 | NCBI |
| *Gluconacetobacter liquefaciens* | WP_114727572.1 | NCBI |
| *Acidiphilium multivorum* | WP_013639477.1 | NCBI |
| *Acidomonas methanolica* | WP_042060361.1 | NCBI |
| *Oleomonas* sp. K1W22B-8 | RJF89425.1 | NCBI |
| *Zymomonas mobilis* subsp. mobilis (strain ATCC 31821 / ZM4 / CP4) | P21907 | UniProt/SwissProt |
| *Treponema pallidum* (strain Nichols) | O83491 | UniProt/SwissProt |
| *Thermotoga maritima* (strain ATCC 43589 / MSB8 / DSM 3109 / JCM 10099) | Q9X0N9 | UniProt/SwissProt |
| *Takifugu rubripes* | P54996 | UniProt/SwissProt |
| *Synechococcus elongatus* (strain PCC 7942) | P29686 | UniProt/SwissProt |
| *Streptococcus pneumoniae* serotype 4 (strain ATCC BAA-334 / TIGR4) | O54537 | UniProt/SwissProt |
| *Solanum tuberosum* | P37830 | UniProt/SwissProt |
| *Schizosaccharomyces pombe* (strain 972 / ATCC 24843) | O59812 | UniProt/SwissProt |
| *Saccharomyces cerevisiae* (strain ATCC 204508 / S288c) | P11412 | UniProt/SwissProt |
| *Rhizobium meliloti* (strain 1021) | Q9Z3S2 | UniProt/SwissProt |
| *Rattus norvegicus* | P05370 | UniProt/SwissProt |
| *Pseudomonas aeruginosa* (strain ATCC 15692 / DSM 22644 / CIP 104116 / JCM 14847 / LMG 12228 / 1C / PRS 101) | O68282 | UniProt/SwissProt |
| *Nostoc punctiforme* (strain ATCC 29133 / PCC 73102) | P48848 | UniProt/SwissProt |
| *Mycobacterium tuberculosis* (strain ATCC 25618 / H37Rv) | P9WN73 | UniProt/SwissProt |
| *Mycobacterium smegmatis* (strain ATCC 700084 / mc(2)155) | A0QP90 | UniProt/SwissProt |
| *Mycobacterium bovis* (strain ATCC BAA-935 / AF2122/97) | P0A585 | UniProt/SwissProt |
| *Mus musculus* | Q00612 | UniProt/SwissProt |
| *Medicago sativa* | Q42919 | UniProt/SwissProt |
| *Macropus robustus* | Q29492 | UniProt/SwissProt |
| *Leuconostoc mesenteroides* | P11411 | UniProt/SwissProt |
| *Kluyveromyces lactis* (strain ATCC 8585 / CBS 2359 / DSM 70799 / NBRC 1267 / NRRL Y-1140 / WM37) | P48828 | UniProt/SwissProt |
| *Homo sapiens* | P11413 | UniProt/SwissProt |
| *Helicobacter pylori* (strain J99 / ATCC 700824) | Q9ZKB2 | UniProt/SwissProt |
| *Helicobacter pylori* (strain ATCC 700392 / 26695) | P56110 | UniProt/SwissProt |
| *Haemophilus influenzae* (strain ATCC 51907 / DSM 11121 / KW20 / Rd) | P44311 | UniProt/SwissProt |
| *Gluconobacter* | Q5FUK8 | UniProt/SwissProt |
| *Escherichia coli* (strain K12) | P0AC53 | UniProt/SwissProt |
| *Escherichia coli* O6:H1 (strain CFT073 / ATCC 700928 / UPEC) | P0AC54 | UniProt/SwissProt |
| *Escherichia coli* O157:H7 | Q8XCJ6 | UniProt/SwissProt |
| *Emericella nidulans* (strain FGSC A4 / ATCC 38163 / CBS 112.46 / NRRL 194 / M139) | P41764 | UniProt/SwissProt |
| *Drosophila yakuba* | Q27638 | UniProt/SwissProt |
| *Drosophila melanogaster* | P12646 | UniProt/SwissProt |
| *Dictyostelium discoideum* | Q557D2 | UniProt/SwissProt |
| *Dickeya dadantii* (strain 3937) | P37986 | UniProt/SwissProt |
| *Cyberlindnera jadinii* | P11410 | UniProt/SwissProt |
| *Cricetulus griseus* | O55044 | UniProt/SwissProt |
| *Chlamydia trachomatis* (strain D/UW-3/Cx) | O84188 | UniProt/SwissProt |
| *Chlamydia pneumoniae* | Q9Z8U6 | UniProt/SwissProt |
| *Chlamydia muridarum* (strain MoPn / Nigg) | Q9PKK8 | UniProt/SwissProt |
| *Ceratitis capitata* | P41571 | UniProt/SwissProt |
| *Caenorhabditis elegans* | Q27464 | UniProt/SwissProt |
| *Buchnera aphidicola* subsp. Acyrthosiphon pisum (strain APS) | P57405 | UniProt/SwissProt |
| *Bos indicus* | Q7YS37 | UniProt/SwissProt |
| *Borrelia burgdorferi* (strain ATCC 35210 / B31 / CIP 102532 / DSM 4680) | O51581 | UniProt/SwissProt |
| *Bacillus subtilis (*strain 168) | P54547 | UniProt/SwissProt |
| *Aspergillus niger* | P48826 | UniProt/SwissProt |
| *Arabidopsis thaliana* | Q9LK23 | UniProt/SwissProt |
| *Aggregatibacter actinomycetemcomitans* | P77809 | UniProt/SwissProt |
